# Long-Term Effectiveness of an Online Self-help Intervention for People with HIV and Depressive Symptoms

**DOI:** 10.1007/s10461-022-03901-4

**Published:** 2022-10-28

**Authors:** Sanne van Luenen, Nadia Garnefski, Philip Spinhoven, Vivian Kraaij

**Affiliations:** 1 Section of Clinical Psychology, Leiden University, Leiden, The Netherlands; 2 Department of Psychiatry, Leiden University Medical Center, Leiden, The Netherlands; 3 Section of Clinical Psychology, Faculty of Social and Behavioural Sciences, Institute of Psychology, Leiden University, P.O. Box 9555, 2300 RB Leiden, The Netherlands

**Keywords:** HIV, Depression, Internet-based intervention, Telemedicine, Cognitive behavioral therapy

## Abstract

The aim of this study was to investigate the long-term effectiveness (3–4 years later) of an online intervention that was previously found to effectively reduce depressive symptoms in people with HIV on the short term. Participants were people with HIV who had participated in the large RCT on the short-term effectiveness of the guided online intervention. The primary outcome measure was depressive symptoms [Patient Health Questionnaire-9 (PHQ-9)] and the secondary outcome measure was anxiety symptoms [Generalized Anxiety Disorder-2 (GAD-2) scale]. Forty-seven participants completed the long-term follow-up. PHQ-9 scores, previously found to have been significantly reduced on the short term, remained low on the long term. GAD-2 scores did not decrease significantly on the short term, however, on the long term, a significant decrease was found. The intervention may not only be effective in lowering depressive symptoms on the short term but also retain the results on the long term.

*Trial registration* International Clinical Trials Registry Platform, NL8448, March 3, 2020.

## Introduction

Depressive symptoms are prevalent among people living with HIV (PLWH) [1, 2]. As these symptoms have an impact on mental health as well as physical health of PLWH, it is important to treat them adequately [3, 4]. For that purpose, the online self-help intervention ‘Living positive with HIV’ had been developed, with the aim of reducing depressive symptoms of PLWH [5]. Online interventions may have benefits compared to face-to-face interventions, such as not having to travel to the psychologist and the possibility to follow the intervention anonymously. ‘Living positive with HIV’ consists of cognitive behavioral therapy (CBT), where psychoeducation is alternated with exercises and assignments. Weekly 15-min telephone coaching is provided to increase motivation and adherence. In a large RCT, it has been found that the guided online intervention was effective in decreasing depressive and anxiety symptoms immediately after the intervention and 3 and 6 months thereafter, compared to an attention-only waiting-list control condition [6]. It is not known yet whether the effects of the intervention are maintained after this period.

Thus far, long-term effects of online and face-to-face psychological interventions for people with depression have rarely been studied [7, 8]. The same applies to the long-term effects of psychological interventions for PLWH to improve mental health [9–11]. However, there is some evidence indicating that the effects of psychological interventions decrease over time; there may be a relapse in symptoms [11–14]. For implementation purposes, it is of utmost importance to know more about the retention of effects of ‘Living positive with HIV’. If an increase of symptoms would be observed over time, an important conclusion could be that relapse prevention activities should be added. It was previously found that booster sessions, follow-up relapse prevention sessions after completion of the treatment, could be beneficial to maintain the effects of psychotherapy for depression [13].

In the current study, the long-term effects of the online intervention Living positive with HIV were investigated (after 3–4 years). The primary aim was to investigate changes in depressive symptoms between post-intervention and long-term follow-up. The secondary aim was to study changes in anxiety symptoms in the same time period. Symptoms of depression and anxiety often co-occur in PLWH [15] and since we measured the short-term effect of the intervention on anxiety symptoms in the RCT, we were also interested in the long-term effects of the intervention on anxiety. The third aim was to explore the needs of participants regarding additional intervention components and/or coaching after completion of the online intervention. These findings may provide further suggestions for the development and implementation of relapse prevention activities after completing the intervention.

## Methods

### Participants

**Fig. 1:**
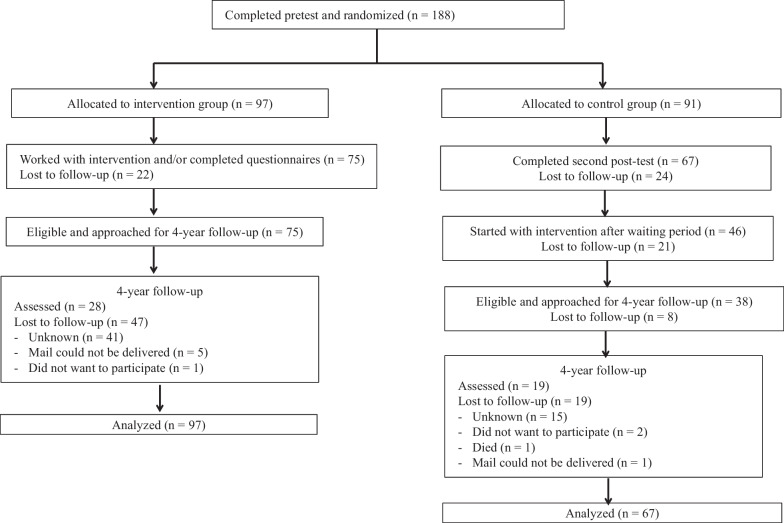
Flow chart of participants in the study

Participants were people who had participated in a large RCT on the short-term effectiveness of the guided online self-help intervention ‘Living positive with HIV’ 3–4 years ago [6]. These participants were people with HIV who had mild to moderate depressive symptoms at the start of the study, aged 18 years and older at the time. The RCT existed of an intervention group and an attention-only waiting list control group (for details see [5, 6]). The participants in the intervention group had worked with the online CBT intervention and received telephone coaching once a week. The control group had received attention only via telephone and they could follow the intervention including coaching after a 5-month waiting period. In the RCT, 188 people had been included. In the current study, all participants of the intervention group (n = 75) and participants of the control group who had started with the intervention after the waiting period (n = 38) were invited to participate. Participants that had dropped-out during the RCT (n = 75) were not approached and this resulted in 113 invited participants (see Fig. 1). All participants in the current study followed the intervention sooner or later, so the control group is no ‘real’ control group anymore. Therefore, the names of the groups were adapted: the intervention group is called intervention group I, and the former control group is called intervention group II.

### Procedure

Participants received an email with information about the follow-up study in January 2020. If they were interested in participating they followed a link to the online questionnaire. At the start of the questionnaire, participants were asked to sign an informed consent. It took approximately 15–20 min to complete the questionnaire.

### Instruments

For the primary aim, the Patient Health Questionnaire-9 (PHQ-9; [16]) was used to measure the severity of depressive symptoms. The PHQ-9 consists of nine questions that can be scored on a scale from zero to three, with higher scores indicating more depressive symptoms. Anxiety symptoms were measured as a secondary outcome with the Generalized Anxiety Disorder-2 (GAD-2) scale [17]. The GAD-2 contains two questions that are scored from zero to three and higher scores point to more anxiety symptoms. The GAD-2 was used in the present study instead of the GAD-7 that was used in the RCT because there was no GAD-7 data available of intervention group II participants on all time points. The correlation between GAD-2 scores and the scores on the other 5 items of the GAD-7 was high (*r* = 0.67–0.81 on the different time points). For the third study aim, open and closed self-composed questions regarding needs were included, e.g. ‘to what extent would you have needed extra components/coaching after the self-help program?’.

### Statistical Analysis

SPSS (version 25) was used to analyze the data and a *p*-value < 0.05 was considered to indicate statistical significance. The analyses for the first and second aim were conducted by intention to treat. For the third aim, only the answers of the participants that completed the questions about their needs could be included in the analyses. The following time-points were included in the analyses: T0 = pretest,^1^ T1 = post-test directly after following the intervention and T2 = long-term post-test (after 3–4 years).

Χ^2^ and ANOVA were used to investigate differences between participants that did and did not complete T2. We did longitudinal multilevel regression analyses [18] using the maximum likelihood estimation to investigate the effect of the intervention on depressive and anxiety symptoms on the short and long term. The analysis handles missing data on the dependent variable in a natural way as it builds a model (with an intercept and slope for each participant) using all available data from all participants, even when only a single measurement is present for a participant. This means participants with missing data were not excluded from the analyses, so all participants present at baseline were included. In particular, by fitting an integrated model to the data from all participants at the same time, one participant’s estimated profile (i.e., intercept/slope) may give information about other participants’ profiles [19]. The analyses on the short-term effects were previously conducted and now repeated, because the sample is not completely the same as before. Before the analyses, we found that the PHQ-9 scores and the GAD-2 scores were not normally distributed and therefore a square root transformation was used. Group (intervention group I and intervention group II) was added as a variable in the analyses. We did not expect differences between groups but included it as a variable in the analyses to check this. Two dummy variables were calculated for time; one variable was used to estimate the short-term effect and one variable to estimate the long-term effect. In the analysis with the PHQ-9, Time and group were included as fixed effects and slopes for time and the intercept as random effects. For the GAD-2 the same analysis was conducted, except that only the intercept was added as a random effect, as this model had a better fit. In addition, a secondary analysis was conducted to investigate the effect of the variable ‘received psychological treatment since program completion’ (yes or no). The same multilevel analysis was conducted with only the long-term effect and the addition of the received treatment variable and the interactions with the other variables. When the interaction Time x ‘received psychological treatment’ was significant, this indicated an effect of received treatment over time. The compound symmetry variance–covariance matrix was used in the analyses.

Cohen’s *d* was calculated to examine the effect size of Time, using the procedure proposed by Morris & Deshon [20, 21]. The SD of the raw scores of intervention group I and the correlation between the pre-test and the post-test of the raw scores were used. Effect sizes were calculated using the estimated values from the longitudinal multilevel regression analyses (which were transformed by squaring them).^2^

Recovery was determined by assessing whether a cutoff for depression (score 10 on the PHQ-9) [22] was reached at T1 and T2. Participants who scored below this cutoff at T0 (no clinical cases) were not included in the assessment, because they had already reached the criterion [23]. Relapse was determined by assessing whether participants scored above the PHQ-9 cutoff while scoring below it in an earlier measurement. The participants that showed no change, i.e. scored below the cutoff on two measurements or above the cutoff on two measurements, are also presented. The raw data were used to determine recovery, relapse and no change.

## Results

### Participants

Forty-seven participants completed the long term follow-up (see Fig. 1). Most participants were males with a high education and a mean age of 50 years (see Table 1). All participants used antiretroviral therapy (ART). No differences in baseline characteristics were found between intervention group I and intervention group II participants and between drop-outs and completers of T2 (see Table 2).

**Table 1:** Demographic characteristics stratified by intervention group

Characteristic	Total sample (n = 47)	*M* (*SD*) or n (%) Intervention group I (n = 28)	Intervention group II (n = 19)
Age (present study)	50.19 (10.21)	51.25 (10.70)	48.63 (9.50)
Sex			
Male	43 (91%)	25 (89%)	18 (95%)
Female	4 (9%)	3 (11%)	1 (5%)
Education			
Low	6 (13%)	4 (14%)	2 (10%)
Medium	15 (32%)	9 (32%)	6 (32%)
High	26 (55%)	15 (54%)	11 (58%)
Marital status			
Married or cohabiting	19 (40%)	9 (32%)	10 (53%)
Single or living without partner	28 (60%)	19 (68%)	9 (47%)
Received psychological treatment since program completion			
Yes	15 (32%)	10 (36%)	5 (26%)
No	32 (68%)	18 (64%)	14 (74%)
Used psychotropic medication since program completion			
Yes	8 (17%)	6 (21%)	2 (11%)
No	39 (83%)	22 (79%)	17 (89%)

**Table 2:** Demographic characteristics stratified by T2 completion

Characteristic	Total sample (n = 188)	*M* (*SD*) or n (%) T2 completers (n = 47)	T2 drop-outs (n = 141)
Age	46.30 (10.63)	46.60 (10.65)	45.40 (10.62)
Sex			
Male	166 (88%)	43 (92%)	123 (87%)
Female	22 (12%)	4 (8%)	18 (13%)
Education			
Low	42 (22%)	7 (15%)	35 (25%)
Medium	77 (41%)	18 (38%)	59 (42%)
High	69 (37%)	22 (47%)	47 (33%)
Marital status			
Married or cohabiting	85 (45%)	19 (40%)	66 (47%)
Single or living without partner	103 (55%)	28 (60%)	75 (53%)
Psychotropic medication			
No	166 (88%)	41 (87%)	125 (89%)
Yes	22 (12%)	6 (13%)	16 (11%)
Time since HIV diagnosis (years)	9.87 (6.58)	9.84 (6.39)	9.96 (7.19)
PHQ-9 score	11.44 (4.50)	10.98 (4.14)	11.59 (4.61)
GAD-7 score	8.86 (4.60)	8.57 (4.49)	8.96 (4.65)

Data is from the baseline measurement of the RCT

*PHQ-9* Patient Health Questionnaire-9; *GAD-7* Generalized Anxiety Disorder-7

### Short-Term and Long-Term Effectiveness of the Intervention on Depressive and Anxiety Symptoms

**Fig. 2:**
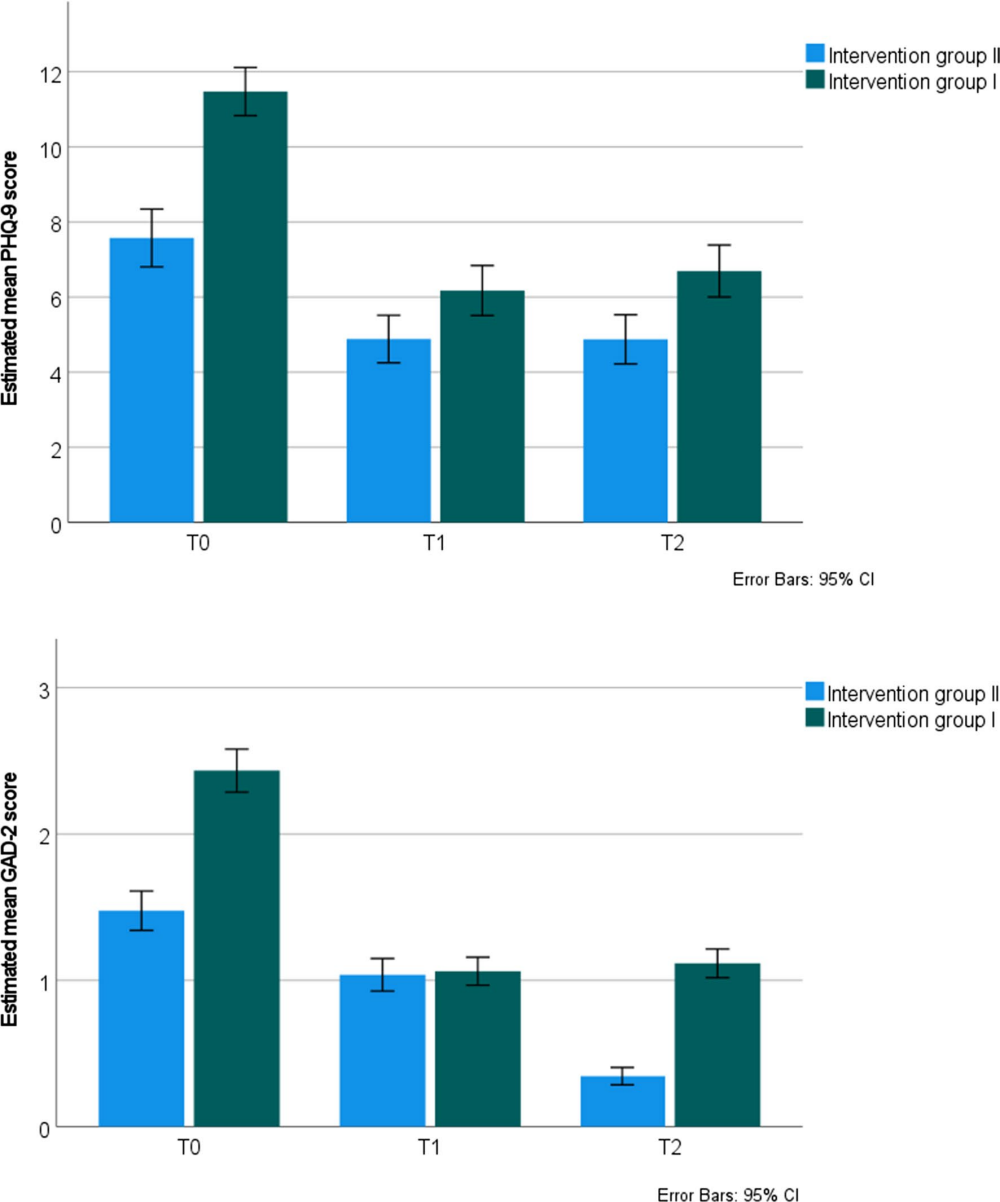
Estimated mean PHQ-9 and GAD-2 scores over time in both intervention groups. *Note GAD-2* Generalized Anxiety Disorder-2; *PHQ-9* Patient Health Questionnaire-9; *T0* pretest; *T1* post-test directly after the intervention; *T2* long-term follow-up (3–4 years)

Estimated means and SDs of the PHQ-9 and the GAD-2 on all time points in both groups can be found in Table 3. For the PHQ-9, previous results of the RCT were confirmed: a significant short-term effect was found; scores decreased from T0 to T1, see Table 4 and Fig. 2. As expected, PHQ-9 scores did not change significantly between T1 and T2, they remained low. In addition, no short-term or long-term Time by Group effect was found for the PHQ-9, intervention group I and II both showed a similar decrease in depressive symptoms over time.

**Table 3:** Estimated means and SDs of the PHQ-9 and the GAD-2 on all time points in both groups

Outcome measure and time point	Intervention group I *M (SD)*	Intervention group II *M (SD)*
PHQ-9		
T0	11.47 (3.18)	7.57 (3.15)
T1	6.18 (3.30)	4.88 (2.59)
T2	6.69 (3.43)	4.88 (2.69)
GAD-2		
T0	2.43 (0.73)	1.48 (0.55)
T1	1.06 (0.47)	1.04 (0.46)
T2	1.12 (0.49)	0.35 (0.24)

*PHQ-9* Patient Health Questionnaire-9; *T0* pre-test; *T1* post-test directly after the intervention; *T2* long-term follow-up (3–4 years); *GAD-2* Generalized Anxiety Disorder-2

**Table 4:** Results of multilevel analyses investigating the effects of the intervention on short term and long term depressive and anxiety symptoms

Outcome measure	Time effect				Time by group effect		
	*b* (SE)	*t*	*p*	Cohen’s *d* (95% CI)	*b* (SE)	*t*	*p*
PHQ-9							
T0 vs T1	− 0.57 (0.18)	− 3.20	0.002	− 0.88 (− 1.11; − 0.65)	− 0.40 (0.21)	− 1.89	0.06
T1 vs T2	0.003 (0.24)	0.01	0.99	0.07 (− 0.15; 0.29)	0.11 (0.30)	0.37	0.71
GAD-2							
T0 vs T1	− 0.20 (0.14)	− 1.40	0.16	− 0.55 (− 0.77; − 0.33)	− 0.34 (0.17)	− 1.99	0.048
T1 vs T2	− 0.45 (0.19)	− 2.39	0.02	− 0.13 (− 0.34; 0.09)	0.48 (0.23)	2.05	0.04

*b* unstandardized coefficient; *t* t-test statistic; *PHQ-9* Patient Health Questionnaire-9; *T0* pre-test; *T1* post-test directly after the intervention; *T2* long-term follow-up (3–4 years); *GAD-2* Generalized Anxiety Disorder-2

For the GAD-2, no significant short-term effect from T0 to T1 was found in this sample, see Table 4. However, scores did decrease significantly on the long term from T1 to T2. Furthermore, a short-term and long-term Time by Group effect was found for the GAD-2. On the short term, intervention group I showed a larger reduction in scores, and on the long term, intervention group II showed a larger reduction in scores. This was contrary to expectations, as we expected that both groups would show the same decrease in anxiety symptoms on the short-term and on the long-term.

The results of the secondary analysis indicated that ‘received psychological treatment since program completion’ had no effect on PHQ-9 and GAD-2 scores over time. The interactions of Time x received treatment were not significant (*p’s* > 0.29).

In Table 5, recovery and relapse rates on the PHQ-9 are presented. Recovery rates are quite high, while relapse rates are approximately 10%.

**Table 5:** N and % of participants that recovered and relapsed

Time points	Recovery	Relapse	No change
T0-T1	15/27 (56%)	2/20 (10%)	30/47 (64%)
T0-T2	18/27 (67%)	2/20 (10%)	27/47 (57%)
T1-T2	6/14 (43%)	3/33 (9%)	38/47 (81%)

Total n = 47

### Needs of Participants After Intervention Completion

Most participants (33 out of 45, 73%) indicated they had worked actively with topics of the intervention, such as starting with activities and relaxation exercises, after completion. However, only six out of forty-five participants (13%) had visited the intervention website after completion of the study. Most participants indicated they did not miss anything in the online intervention. Twelve participants (27%) indicated that an extra lesson or coaching after the intervention was needed not at all, 12 participants (27%) indicated it was hardly needed, 14 (31%) indicated a little, and 7 (16%) indicated quite needed. The opinions on the content of an extra lesson or coaching after the intervention differed a lot. For example, some participants indicated they would have preferred reminders, updates, or a repetition of the intervention. Others indicated that check-ups by the coach or contact with peers would be a good addition.

## Discussion

The current study aimed to investigate the long-term effects of the online intervention ‘Living positive with HIV’. In addition to the reduction in depressive and anxiety symptoms on the short term that was previously found [6], symptoms remained low 3–4 years after intervention completion. This confirmed that the intervention may also be effective in the retention of low depressive and anxiety symptoms in PLWH on the long term. The results apply to participants that did and did not receive psychological treatment since program completion. Furthermore, most participants appreciated the intervention as it was and did not express a need for an extra lesson or extra coaching after the intervention.

Not much research into the long-term effects of online and face-to-face interventions for depression has been done, but most previous studies found an increase in symptoms over time [11–14]. Previous studies that used booster sessions to retain the effects of the intervention had in general better treatment results on the long term than studies without these booster sessions [13]. The intervention in the current study did not have booster sessions, but many participants that completed the long-term follow-up indicated they had actively worked with topics of the intervention after completion of the study. For example, they started with activities, did the relaxation exercises, or attempted to change their negative thoughts. Although the intervention website had not been visited by many participants after the study, they still were able to apply the learned techniques in their daily life. This may be the reason that depressive and anxiety symptoms remained low 3–4 years later. Since many participants that completed the long-term follow-up indicated they still worked on the topics of the intervention after study completion and only a minority of participants showed a relapse in symptoms, it may not be necessary to add booster sessions to the intervention. However, as many participants of the RCT did not complete the long-term follow-up, we do not know whether this also applies to the dropouts. More research into the effect of booster sessions and for whom it would be necessary may be conducted.

We also investigated the differences between intervention group I and intervention group II, which was the former control group that followed the intervention after the waiting period. We did not expect differences between both intervention groups. However, intervention group II had significantly lower pretest depression and anxiety scores than intervention group I. Intervention group II participants had been on the waiting list before they started with the intervention and had already received (minimal) attention during the waiting period. Participants had appreciated the attention and that may have led to a reduction in symptoms already [24], before they started with the intervention. Furthermore, no differences were found between the intervention groups in the reduction in PHQ-9 scores in the short term and long term; both groups showed a similar decrease in symptoms. However, a difference between groups over time was found for the GAD-2; participants in intervention group I showed a larger reduction in scores on the short term, and participants in intervention group II showed a larger reduction in scores on the long term. The lower pretest scores of participants in intervention group II may play a role, which may be due to the use of data of the second post-test of the RCT (after being on the waiting list) as the baseline for intervention group II participants, in contrast to the ‘real’ baseline data that was used for intervention group I participants.

The current study has some limitations. First, there were two intervention groups, but no control group. Therefore, the long-term effects of the intervention groups could not be compared with a control group that did not follow the intervention. For future research, differences in long-term effects of eHealth interventions between intervention and control group participants should be investigated. In addition, both intervention groups were not completely the same, as intervention group II participants had been on the waiting list before. Second, the proportion of participants that completed the long-term follow-up was small, only 0.42. A large non-response had been expected because the original RCT was conducted 3–4 years ago. The analysis can handle missing data very well, but the data should be missing completely at random and it is not clear if this is the case for all missings. However, we did not find differences in baseline characteristics such as age, sex and symptom scores between responders and non-responders of T2, which indicates that the results may be generalized. Though, there may be differences between the groups that we are not aware of and the follow-up sample is small. More research is needed to confirm these findings for the population of PLWH. Lastly, the Center of Epidemiologic Studies Depression Scale (CES-D, [25]) that we used in the RCT was not included in the current study, to keep the questionnaire short and because the results of the PHQ-9 and the CES-D were comparable in the RCT. Furthermore, the GAD-2 was used in this study, instead of the longer GAD-7. Therefore, the results of the RCT cannot be adequately compared to the results of the current study.

More research should be conducted into the long-term effects of online interventions for depression, as not much is known on this topic. Furthermore, the effect of booster sessions and reminders and for whom it would be necessary may be investigated in future research, by including one group that receives booster sessions and/or reminders and one group that does not receive that. In addition, predictors and moderators of treatment effects on the long term should be investigated to improve knowledge of which treatment may be best suited for which patient over time.

In conclusion, the results suggest that the online intervention ‘Living positive with HIV’ may not only be effective on the short term, but also on the long term. In general, there was no relapse of depressive and anxiety symptoms 3–4 years after completion of the study in a small sample. In addition, the results apply to participants that did and did not receive psychological treatment since program completion. Many participants indicated they actively worked with intervention topics after the study had ended. Currently, the online intervention with coaching has been implemented for PLWH in the Netherlands, which seems justified because of the positive effects on the short term and on the long term.

## Data Availability

The datasets generated and/or analyzed during the current study are available from the corresponding author on reasonable request.

## Footnotes

1 Intervention group II had started 5–6 months later with the intervention than intervention group I in the original RCT because they were on the waiting list first. Therefore, the data from the second post-test of the RCT was used as the pre-test for intervention group II participants, as the second post-test was administered just before the start of the intervention.

2 The effect size was calculated differently in the paper concerning the RCT. We could not use that method because we use square root transformed scores now.
